# Properties of Biocomposites Made of Extruded Apple Pomace and Potato Starch: Mechanical and Physicochemical Properties

**DOI:** 10.3390/ma17112681

**Published:** 2024-06-02

**Authors:** Adam Ekielski, Tomasz Żelaziński, Ryszard Kulig, Adam Kupczyk

**Affiliations:** 1 Department of Production Engineering, Institute of Mechanical Engineering, Warsaw University of Life Sciences, Nowoursynowska 164, 02-787 Warsaw, Poland; adam_ekielski@sggw.edu.pl (A.E.); adam_kupczyk@sggw.edu.pl (A.K.); 2 Department of Food Engineering and Machines, University of Life Sciences in Lublin, 20-950 Lublin, Poland; ryszard.kulig@up.lublin.pl

**Keywords:** extruded apple pomace, biocomposites, starch potato, mechanical properties, thermal analysis

## Abstract

This paper presents research results on biocomposites made from a combination of extruded apple pomace (EAP) and potato starch (SP). The aim of this work was to investigate the basic properties of biocomposites obtained from extruded apple pomace reinforced with potato starch. The products were manufactured by hot pressing using a hydraulic press with a mould for producing samples. The prepared biocomposites were subjected to strength tests, surface wettability was determined, and a colour analysis was carried out. A thermogravimetric analysis (TGA), Fourier transform infrared spectroscopy (FTIR), and cross-sectioning observed in a scanning electron microscope (SEM) were also performed. The obtained test results showed that the combination of apple pomace (EAP) and starch (SP) enabled the production of compact biocomposite materials. At the same time, it was found that each increase in the share of starch in the mixture for producing biocomposites increased the strength parameters of the obtained materials. With the highest share of starch in the mixture, 40%, and a raw material moisture content of 14%, the material had the best strength parameters and was even characterised by hydrophobic properties. It was also found that materials with a high content of starch are characterised by increased temperature resistance. The analysis of SEM microscopic photos showed well-glued particles of apple pomace, pectin, and gelatinised starch and a smooth external structure of the samples. Research and analyses have shown that apple pomace reinforced only with the addition of starch can be a promising raw material for the production of simple, biodegradable biocomposite materials.

## 1. Introduction

In the era of concern for the natural environment around us, finding ways to reduce the extraction of fossil fuels, from which traditional plastics are also made, is crucial. This can be supported by the production of modern biodegradable materials based on plant raw materials, which in many cases can provide an alternative to plastics [1]. Such materials are appreciated by the public; previous research has confirmed their usefulness in many areas, e.g., packaging, disposable tableware, car upholstery components, structural boards and beams, etc. [2,3,4,5]. Various plant-based raw materials rich in cellulose, pectin, and proteins can be used to manufacture such materials, as research has confirmed [6]. This opens up new possibilities for the application of such plant raw materials, the management of which is difficult or economically unjustifiable. Among such raw materials is apple pomace, which is a massive by-product of processing apples into juice. It is estimated that more than 20 million tonnes of apple pomace are produced worldwide each year [7]. Factors supporting their use include large-scale production and a chemical composition that is favourable, both for the production of food and non-food raw materials [8]. However, the chemical composition may vary, both by apple variety and by the region where they are grown, which may limit their further processing. On average, apple pomace contains cellulose (7–22%), lignin (15–20%), starch (14–17%), pectin (4–14%), and small quantities of protein [9,10,11]. Due to its pectin, sugar, and insoluble fibre content, much of it is used for the production of nutritional supplements or animal feed [12]. Pomace can also be processed into pellets and used as fuel [13,14]. Apple pomace is also used as a natural organic fertiliser [15]. The remainder of the apple pomace produced is usually composted (intensive fermentation processes take place in this raw material), which is not beneficial to the environment and, in particular, to groundwater [16,17]. This particularly argues for an even wider use of apple pomace, especially for non-food purposes.

One interesting area in which apple pomace could potentially be used is the packaging industry, which is currently focused on the search for innovative biodegradable materials. Interest in pomace as feedstock for biodegradable materials is still low and is mainly limited to the use of pomace as an additive of a few percent. Studies have been carried out, among others, on biodegradable active film packaging in which apple pomace or apple pomace extracts acted as an antioxidant released during food storage [18,19]. Attempts have also been made to enrich TPS starch with apple pomace [20]. Overall, researchers mainly point to the positive aspects of incorporating apple pomace (and other types of pomace) into a polymer matrix [21,22]. Reports from the scientific literature indicate that the pelletisation of apple pomace is feasible, so it is possible to thicken apple biomass to obtain a compact structure [23]. Other authors report that increasing the functionality of similar products is possible by obtaining pectin bonds (gel) in the product, but this requires strictly defined heating as well as cooling conditions. The same authors also claim that the pectin contained in apple pomace enables the formation of membranes and allows mixing with other polymers. It has also been found that monosaccharide content in the pomace allows pomace molecules to stick together, but the resulting material is hygroscopic and, consequently, can quickly lose its mechanical properties in the presence of water [24].

Literature review confirms that apple pomace can be used as an additive in the manufacture of biocomposites. However, the literature lacks precise information on how and under what conditions the particles of this raw material are combined during compaction. Based on data from the literature, it also appears that one of the main problems in producing a biodegradable material from apple pomace is obtaining adequate strength of such a material. The authors of [25] argue that this is usually related to poor bonding quality between the particles that make up the pomace. This is particularly the case when biocomposites are produced by pressure compacting. For this reason, many authors attempt to increase press force, which improves strength properties of the materials obtained, allowing more efficient use of van der Waals forces in the material being pressed [26]. However, in order to achieve the right mechanical properties, the use of van der Waals forces alone requires adequate material fragmentation and relatively high contact forces, up to 500 MPa [23], to allow material molecules to approach one another sufficiently. When such contact forces are applied, unit energy required to produce the material increases significantly, which is mainly related to intermolecular friction [27].

Therefore, the addition or activation of natural adhesive substances contained in plant products in order to naturally reinforce the materials obtained seems to be a more promising method. The extrusion process, which, as a result of temperature and pressure, can modify the structure of both starch-based and non-starch-based raw materials, may be used for this purpose [28]. Such experiments have not been performed so far and, therefore, they fill the gap in biocomposite research. Moreover, the extrusion process was not used to pre-prepare apple pomace for the production of biocomposites.

In this context, the use of the extrusion process for processing apple pomace makes a new contribution to the development of innovative biocomposites, and the results obtained go beyond the current state of the art. From a utilitarian point of view, it is also beneficial to use the extrusion process for several important reasons: reducing the water content of wet pomace, reducing water activity, and stopping microbiological processes (including, for example, deactivation of possible moulds) in apple pomace [29]. These properties are extremely important for products that come into contact with food. Moreover, the processed pomace can be stored and can also be a promising raw material (or additive) for the production of innovative biocomposites on a large scale—very necessary to replace some plastic products (e.g., packaging, disposable tableware, or even construction elements) [28]. In the case of apple pomace, pectin is the most promising gluing component. Other interesting components are lignin and cellulose, found in the composition of apple pomace, which can also reinforce biocomposites. Despite their presence in apples, studies carried out to date have shown relatively low strength of materials made exclusively from apple pomace [30]. Therefore, a promising addition could be the introduction of starch as a reinforcing substance (natural polymer, commonly found in nature). In its crystalline form, starch forms an amorphous structure under the influence of temperature and in the presence of a plasticiser, which significantly facilitates moulding, while acting as a binding agent as well. Depending on the type of plasticiser used and the ratio of amylose to amylopectin contained in the starch, different properties of processed starch can be obtained [31]. Research into the use of starch as an additive in the manufacture of apple pomace films was carried out by Gustafsson et al. [32]. Unfortunately, their research is difficult to relate to solid materials. The production of films takes advantage of the phenomenon of the dissolution of components contained in pomace. In the process of pressure moulding, the bonding of molecules by using pressure and temperature forces is exploited.

The materials produced in the research fit into the “zero waste” ideology, which strives to reduce the pollution of the surrounding environment. Unfortunately, despite many studies, the physical, chemical, and mechanical parameters are still not satisfactory compared to plastics [8,28]. On the other hand, many plastics are oversized (e.g., too durable). Research on the basic properties of newly created biodegradable materials (especially strength, absorption, and thermogravimetric parameters) in the context of their further use is therefore very justified and necessary. Given the above, the aim of the present study was to investigate the basic properties of biocomposites obtained from extruded apple pomace reinforced with potato starch. The aim of this work was also to identify the possibility of using extruded apple pomace as a basic raw material for the production of biocomposites.

In this study, starch acted as an additional binder, strengthening the structure of the obtained biocomposites. In the longer term, the results obtained may contribute to the production of new biodegradable utility products.

## 2. Materials and Methods

### 2.1. Materials

The research material was wet apple pomace (moisture content: 60% (±2%)) purchased on the local market (Greenherb, Łancut, Poland). Before being used, the pomace was stored under refrigeration at a temperature of +1 °C. The basic chemical composition of the pomace was raw ash at 2.4%, total protein at 5.47%, crude fat at 3.92%, and crude fibre at 58.25% of dry mass. (SP) potato starch (Superior) was purchased from a local producer (Potato Industry Company, Trzemeszno, Poland). The primary chemical composition of the starch was as follows: crude ash at 0.4%, total protein at 0.06%, crude fat at 0.05%, amylose at 21.2, and amylopectin at 79.1% of dry mass.

#### Pre-Treatment of Apple Pomace and Biocomposite Production

Apple pomace extrusion process: The pomace was rinsed in water at 16 °C to remove soluble compounds. After draining excess water in a piston press, moisture content of the pomace was 50% (±0.2%). This was the optimal moisture content determined experimentally, which enabled pre-treatment in the extrusion process (moreover, achieving this moisture content did not require the use of energy-intensive devices). This is an important stage of the process due to the repeatability of the results. The pomace with this moisture content was pre-treated in a high-temperature extruder. The purpose of this method was to pregelatinise the starch and the gelation of pectin contained in the pomace and to homogenise the apple pomace. A utilitarian effect was also the reduction in the moisture content of the raw material. A modified single-screw extruder (Insta Pro 600, manufacturer: Insta Pro, Grimes, IA, USA) with an L:D ratio of 12:1, retrofitted with electric heaters, was used in the tests. Temperature distribution in the extruder was, respectively, 80/80/100/120 °C. The head with the extruder matrix had the highest temperature of 120 °C. The extruder head uses two round nozzles with a diameter of 8 mm and a length of 70 mm. A knife cutter was used on the extruder matrix. The rotational speed of the extruder screw was 550 RPM. The raw material flow rate in the extruder was 80 kg·h^−1^, +/−0.2 kg.

Drying of pomace: After the extrusion process and cooling of the samples (before measurement, they were cooled on a perforated surface), the moisture content in the extrudate was 17.5% (+/−0.2%). The resulting extruded (EAP) apple pomace was dried in a convection dryer to a moisture content of 10%. For research purposes, potato starch was also dried (or moistened, if necessary) to a moisture content of 10% (+/−0.2%).

Grinding and preparing mixtures: The dry material was crushed in a beater shredder, Bąk H11/3 (manufacturer: Agromet, Jawor, Poland), equipped with 1 mm hole diameter sieves. The final material was obtained by sieving the dry apple pomace in a sifter through 0.5 mm hole sieves. This was to eliminate large particles that could interfere with the structure and quality of the obtained biocomposite. The moisture content in apple pomace (EAP) and potato starch was measured using a laboratory moisture analyser (MA 210 R, manufacturer: Radwag, Warsaw, Poland).

**Figure 1:**
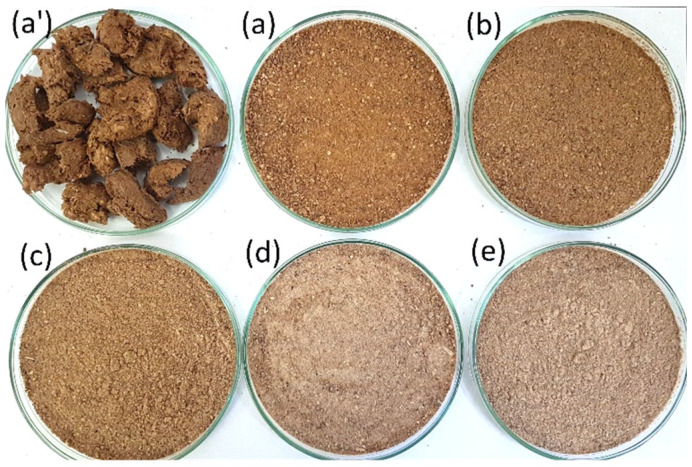
Raw materials used to prepare biocomposite samples: (**a’**)—apple pomace after the extrusion process; (**a**–**e**)—apple pomace after grinding and mixing with potato starch; (**a**)—0, (**b**)—10, (**c**)—20, (**d**)—30, (**e**)—40% addition of potato starch (SP). Moisture content in the raw material: 12%.

The mixes used in the tests were prepared in a ribbon mixer by adding water to dry matter to obtain a raw material with a moisture content of, respectively, 10, 12, and 14% (±0.2%). A detailed test plan showing the raw material composition of the biocomposites is shown in Table 1 (percentage values refer to total weight). Figure 1 also includes images of extruded pomace and raw materials after grinding and mixing the addition of potato starch. The material thus obtained was conditioned in a sealed container for 1 h at room temperature.

**Figure 2:**
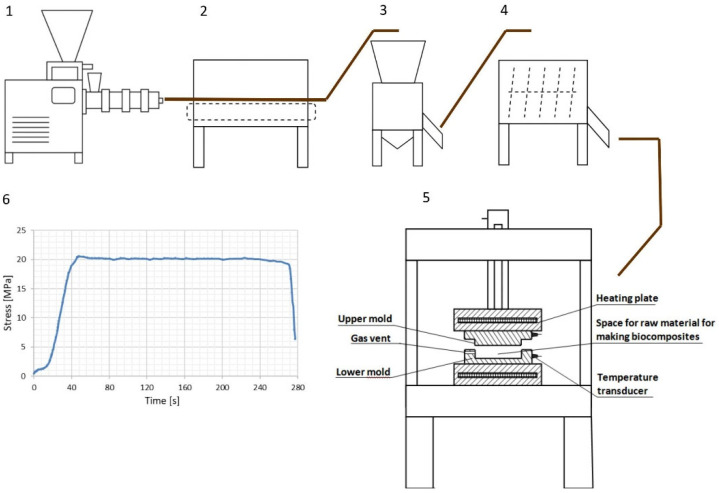
Technological diagram for preparation of raw material and moulding of biocomposites: 1—single-screw extruder, 2—convection dryer, 3—hammer mill, 4—band mixer, 5—hydraulic press with a matrix for forming samples, 6—plot of the stress curve of a pressed composite strength.

Biocomposite production: The material obtained in this way was then compacted by hot pressing on a test stand, which consisted of a hydraulic press (FR—5014, manufacturer: Farys, Rzepin, Poland) with a maximum pressure of 150 kN. Electrically heated plates were installed on the piston pin and at the bottom of the press, with heaters with a total power of 1.6 kW installed inside the plates. The temperature was controlled by heater controllers with the accuracy of ±0.1 °C. Plate wall thickness was 15 mm (±0.1 mm) on both sides of the heater. A metal mould was inserted between the plates, which allowed an 81 cm^2^ sample to be moulded/compressed to a thickness of 5 mm (±0.1 mm) (Figure 2).

The weight of a single sample poured into the mould at a time was 80 g. The value of stress during the pressing process that was applied during the tests was 20 MPa. The biocomposite moulding tests were carried out at the temperature of 140 °C (±0.1 °C). The pressing time for each sample was 4.5 min. We selected the temperature, stress, and time based on preliminary research. The raw material used in the research was very sensitive to changes in process parameters (e.g., it had a tendency to split, burn, etc.); therefore, we selected the pressing parameters (stress and time) experimentally. Similar conditions were also used in article [33].

The above parameters enabled proper compression only of biocomposites from extruded apple pomace (EAP). With the assumed process parameters, it was not possible to properly produce samples of biocomposites from apple pomace that had not been pre-treated in the extruder (such material was too intensive, delaminated after opening the die, and poor to control the flow of material during forming). Therefore, for the purposes of this article, we did not produce and compare samples of biocomposites made from raw apple pomace.

All biocomposite samples intended for testing were stored in a controlled atmosphere (temperature at 25 °C and relative humidity (RH) at 40% in the KBK-30 WAMED climatic chamber, Warsaw, Poland).

### 2.2. Mechanical Properties

**Figure 3:**
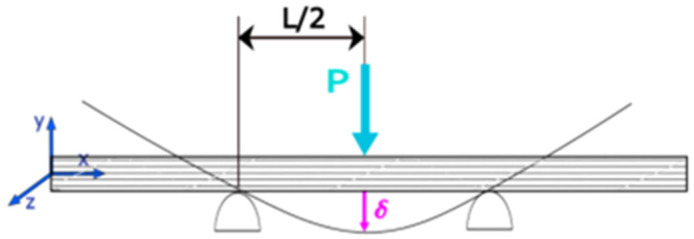
Bending strength tests.

Strength tests were carried out in accordance with ISO 178:2019 [34]. The standard describes testing of mechanical properties of plastics. The basic assumption underlying this standard is homogeneity of the material. In order to carry out the strength tests, the AXIS 500 stress testing device with an FA 500 N load cell was used (manufacturer: AXIS, Gdansk, Poland). Samples in the form of a 5 × 15 × 90 mm rectangular beam were placed on two supports (distance between the supports: 40 mm), and then subjected to a static bending load (Figure 3). The bending force was concentrated in the sample symmetry axis. The strength test was continued until the sample broke. The bending head displacement rate was calculated according to the following equation, Equation (1):

(1) $$v=\frac{al^{2}}{6h}=\frac{0.01\cdot 40^{2}}{6\cdot 5}=0.53\;{(mm\cdot min}^{-1})$$

where *a* is the deformation rate of the outer fibre (mm × min^−1^), *l* is the distance between supports, and *h* is the sample thickness.

The test was completed when the sample deformation reached 5%.

Based on the tests performed, the bending strength and Young’s modulus were determined.

Bending strength *R_g_* as the highest stress value was calculated according to the following relationship (2, 3, 4, 5, 6):

(2) $$R_{g}=\frac{F_{g}l}{{4W}_{g}}$$

where

*F_g_*—bending force (the greatest force recorded during bending);

*l*—distance between supports (constant value in our case);

*W_g_*—bending strength index;

or *W_g_* for a beam with a rectangular cross-section:

(3) $$W_{g}=\frac{{bh}^{2}}{6}$$

where

*b*—beam width;

*h*—beam height.

Next, Young’s modulus (*YM*) was calculated using the following relationships:

(4) $$YM=\frac{{F_{0.2}l}^{3}}{{48f_{0.2}I}_{y}}$$

where

*F*_0.2_—bending force corresponding to a deformation equal to 0.2% of the total deformation of the extreme fibres of the beam;

*l*—distance between supports (constant value in our case);

*f*_0.2_—deflection arrow corresponding to a deformation equal to 0.2% of the total deformation of the extreme fibres of the beam;

or *f*_0.2_ for a beam with a rectangular cross-section:

(5) $$f_{0.2}=\frac{l^{2}\varepsilon }{6h}$$

*ε* = 0.2%

*I_y_*—moment of inertia of the cross-section;

*I_y_* for a beam with a rectangular cross-section:

(6) $$I_{y}=\frac{{bh}^{3}}{12}$$

### 2.3. Density

Apparent density tests of biocomposites (including sample preparation) were performed in accordance with the ASTM D 792 standard [35].

### 2.4. Water Contact Angle

The wetting angle was measured using the sitting drop method. In this method, the wetting angle was determined by analysing the drop shape (the internal angle of inclination of the tangent to the horizontal projection of the drop outline at its point of contact with the tested surface). The shape of the drop was recorded with an A2500-14uc digital camera, 5 Mpix (16.2 Mpix matrix), with 18–105 mm lens (manufacturer: Basler, Ahrensburg, Germany) attached to an adjustable measuring table. Drops of distilled water with the volume of more than 15 µL were applied from a syringe fixed in a holder onto the surface of the biocomposite to be analysed. The same height of syringe attachment was maintained when delivering the droplet onto the biocomposite surface. The choice of drop size was based on the need to minimise drop spreading under gravitational forces, which underestimates the wetting angle. Images were taken 1 s after the drop was placed. The wetting angle was measured by subjecting the drop images to an analysis using Autodesk Autocad Mechanical 2019 software, product version: 23.0.46.0.2.5 (manufacturer: Autodesk, San Rafael, CA, USA).

### 2.5. Colour Analysis

Colour analyses were carried out on the basis of images of the sample surface taken with an STX OPTA-TECH stereo microscope equipped with a 5-megapixel (Mpix) camera, (manufacturer: OPTA-TECH, Warsaw, Poland). The area photographed was illuminated with a circular LED illuminator with the colour temperature of 7000 K. The camera was calibrated before photographing by performing the white balance on a Minolta reference plate, no. 1863310. The colour tests were performed using CorelDRAW X7 Version 17.1.0.572 software (manufacturer: Corel Corporation, Ottawa, ON, Canada), where the L*a*b* space was selected for the analysis of colour changes. The individual letters stand for the following: L*—brightness; a*—colour from green to magenta; and b*—colour from blue to yellow.

### 2.6. Scanning Electron Microscope (SEM)

A HITACHI S-3400N (manufacturer: Hitachi, Tokyo, Japan) scanning electron microscope (SEM) (manufacturer: Hitachi, Tokyo, Japan) was used to take images of the fractures and the outer surface of the samples. The following parameters were set in the microscope: accelerating voltage of 20 kV and low vacuum of 70 Pa.

### 2.7. Thermogravimetry Analysis (TGA) and Derivative Differential Thermal Analysis (DTA)

The samples tested were subjected to a thermogravimetric analysis using TGA and DTA methods. The tests were carried out on a TGAQ50 V20. 13. Build 39 thermogravimetric analyser (manufacturer: TA Instruments, New Castle, DE, USA). The TGA tests were carried out for analytical samples weighing app. 10 mg. Sample mass changes were measured in an inert atmosphere (nitrogen flow rate (40 mL·min^−1^)). Temperature change took place at a rate of 10 °C × min^−1^. The temperature change range was from 30 to 795 °C. As a result of the performed analyses, a so-called thermogravimetric curve (TGA) was obtained in the following system: sample mass—temperature. The differential thermal analysis (DTA) was carried out in parallel to the TGA measurements. As a result, the derivative of the thermogravimetric curve of mass versus temperature was obtained. In this way, the temperature-dependent change in the reaction rate is shown. The maxima on the differential curve define the points at which the rate of temperature decrease due to reaction progress and the rate of temperature increase are maximum. The application of the DTA method facilitates the analysis of thermal effects including the determination of the temperature of onset and extremes of the thermal effect. A summary of the TGA/DTA curves for each sample is shown in the chart.

### 2.8. FTIR Infrared Spectrum Analysis

The (FTIR) infrared spectrum analysis was performed with the FTIR Nicolet 8700 spectrophotometer (manufacturer: Thermo Fisher Scientific, Waltham, MA, USA). The spectra were recorded with the resolution of 2 cm^−1^ in the range of 400–4000 cm^−1^. The tests were performed at room temperature.

### 2.9. Moisture Absorption

To perform MA moisture absorption tests, biocomposite samples with dimensions of 5 × 15 × 90 mm were used and dried in a convection dryer to constant humidity. Samples prepared in this way were placed in the KBK-30 climatic chamber (manufacturer: WAMED, Warsaw, Poland) and the following climatic conditions were set: relative air humidity inside the chamber (RH)—50, 75, and 90%; temperature—25 °C; and time—24 h. First, the initial mass was determined, *W*_0_, for each sample, and then mass measurements were made every hour. The percentage of moisture absorption was calculated using equation

(7) $$Moisture\;absorption=\frac{W_{t}-W_{0}}{W_{0}}\times 100\%$$

### 2.10. Statistical Analysis

The results obtained from the empirical data were statistically processed using the following statistical software: StatSoft, Inc. (Tulsa, OK, USA, 2013), STATISTICA, version 13.1 (TIBCO Software Inc., Palo Alto, CA, USA) (Table 1). For statistical analyses, the DOE experiment planning module (compositional master plans) was used, where significance was determined by the ANOVA test. The confidence level that indicated significant differences between samples was set at 95% (*p* < 0.05). Next, three-dimensional plots (response areas) were made from the data obtained. The above empirical test plan was applied to present the results: bonding strength, Young’s modulus, wetting angle, and L*a*b* colour parameters. In order to present results of the TGA and FTIR analyses, extreme measurement points from the model were selected (moisture: 10 and 14%). The tests were performed in 3 replicates.

## 3. Results

### 3.1. Bending Strength and Young’s Modulus

**Figure 4:**
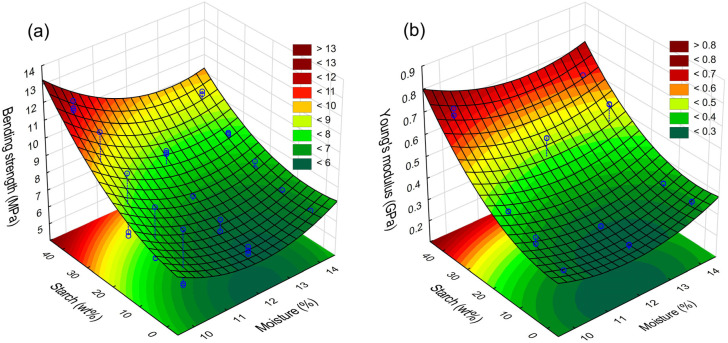

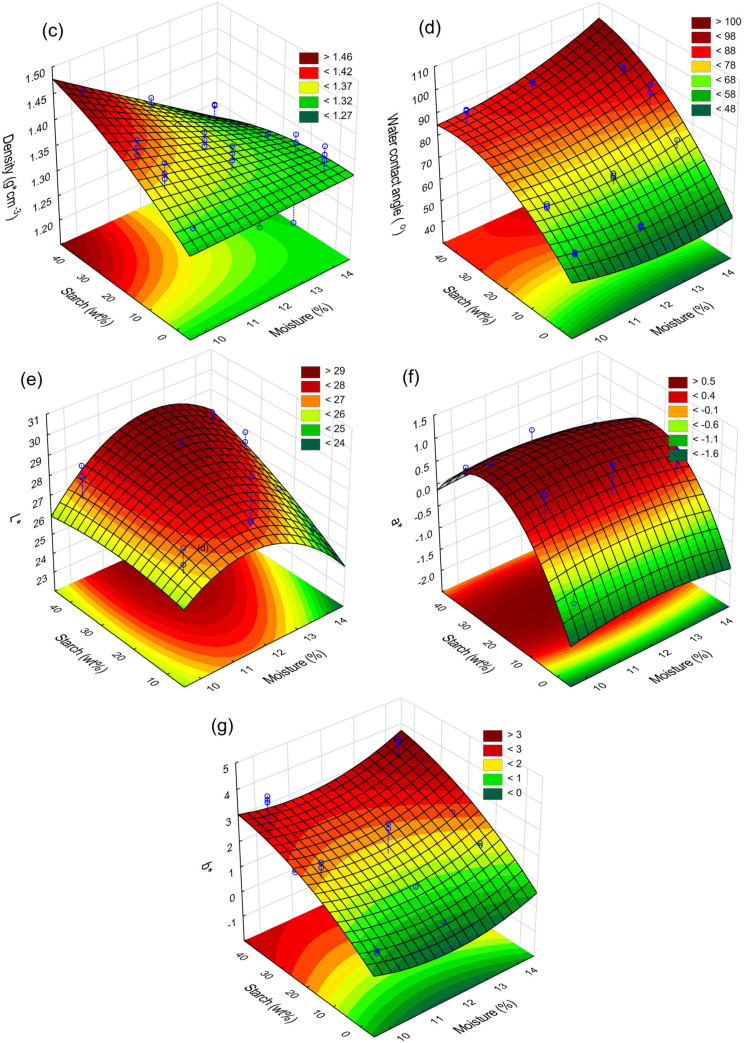
Influence of potato starch (SP) addition and raw material moisture content on parameter changes: (**a**)—bending strength, (**b**)—Young’s modulus (YM), (**c**)—density, (**d**)—water contact angle, (**e**)—colour component L*, (**f**)—colour component a*, (**g**)—colour component b*.

Strength tests showed that it was possible to reinforce (EAP) apple pomace biocomposites with (SP) potato starch. The results obtained show that an increase in the share of (SP) starch in the mixture resulted in an increase in bending strength of the materials obtained from 6.1 to 12.6 (MPa). Furthermore, it was found that an increase in the moisture content of the raw material also caused an increase in strength, with these changes being noticeable for the sample with a 100% content of apple pomace and for samples with up to 30 wt% starch (Figure 4a). The significance of the above data was confirmed by results of the ANOVA in Table 2. The increase in strength of the obtained materials can be attributed to the gelling properties of (SP) potato starch, which, in the presence of polysaccharides (e.g., pectin, inulin), further increased its viscosity properties; this phenomenon was observed by [36]. Other researchers have observed that heat treatment of, e.g., pectin, increases the hardness and gumminess of gels [37]. This observation also justifies the purpose of treating apple pomace in the extrusion process, which interacts favourably with starch during pressing (compression) of biocomposites. The use of (EAP) extruded apple pomace in this study, therefore, may increase strength of the materials obtained, particularly those with an addition of (SP) starch. Studies by other authors also indicate that the crosslinking properties of both starch and pectin also influence the increase in strength [38]. Furthermore, in addition to the presence of pectin, apple pomace is also rich in lignocellulosic compounds, dietary fibre, and other substances, which, under elevated temperature conditions, combine with the matrix of gelled starch to reinforce the final structure of the product during hot pressing of the material. This further justifies the increase in strength as the proportion of (SP) starch in the biocomposite mixture increases. A surprising observation is the highest strength of the biocomposite with a starch share of 40%, with a 10% moisture content in the raw material. Despite obtaining the best strength parameters, in this case, the starch may not have been fully melted and acted mainly as reinforcement (melting starch is commonly defined in the scientific literature as the transition from a crystalline to an amorphous state) [29]. Melted starch was observed in SEM microscopic examinations. In this case, strengthening of the material may also have been related to the greater influence of Maillard reactions, which positively influence the crosslinking of starch and proteins contained in the apple pomace [39]. This may also explain the highest resilience modulus (0.72 GPa), which was also obtained at a 40 wt% (SP) starch share in the mixture and raw material moisture content of 10% (Figure 4b). However, one can conclude that Young’s modulus increased almost linearly with increasing the addition of starch. An interesting observation is that the highest Young’s modulus (YM) values were also achieved at 10% and 14% raw material moisture content, which may indicate that in these cases, two different phenomena may have taken place that affected the resilience and strength of the biocomposites obtained. The first is the melting of starch and subsequent stiffening of the matrix/carcass of the biocomposite after cooling. The other is strengthening of the carcass by granules of unmelted starch due to insufficient moisture content in the raw material.

When comparing the results obtained to studies by other authors, one can conclude that the parameters obtained are sufficient for the manufacture of selected products that do not require high loads, e.g., plates, cups, saucers, packaging, decorative elements, etc. In agriculture, such biocomposites can be used to produce flower pots, multiplatons, saucers, etc. In this case, large-scale plastic flower pots could be replaced with ecological single-use products. For example, in the study by Lang et al., 2021 [40], pressed biocomposites had a bending strength between 8 and 14 MPa and an elastic modulus of 0.45 and 0.55 GPa. In turn, in the work of Picard et al. (2020) [25], apple pomace-based biocomposites with reinforcing additives had a bending strength of 36–55 MPa and a Young’s modulus (YM) of 0.85 to 1.9 GPa. In light of the literature, the results obtained in this research seem satisfactory. Despite this, in order to expand the possibilities of biocomposites, it seems beneficial to increase the strength parameters to at least 20–40 MPa, as indicated by the work of Rahman et al., 2021 [41]. Yet, this requires the use of more advanced additives, which was carried out in the work of Picard et al. (2020) [25]. Among the strength tests, the bending strength was chosen because this parameter best reflects the forces acting in products made by hot pressing. Moreover, any products made of such materials, e.g., cups, trays, plates, etc., are most often subject to bending stresses.

### 3.2. Density

**Figure 5:**
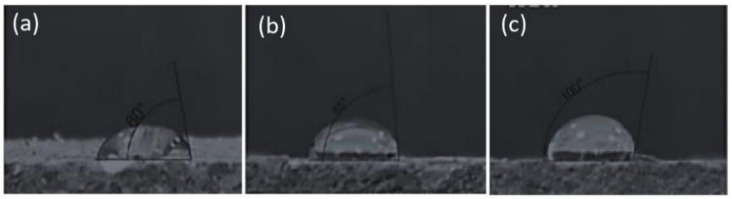
Images of exemplary samples used to test the water contact angle of the surface of biocomposites with the addition of SP 40% starch, at different mixture humidity: (**a**) 10%, (**b**) 12%, and (**c**) 14% moisture.

Density tests for biocomposites are shown in Figure 4c. Analysing the obtained results, it was found that increasing the starch content in the biocomposites (at a moisture content of 10 and 12 wt%) in the mixture resulted in an increase in the density of the materials produced. At the same time, the highest density was observed in biocomposites with the addition of 40% (SP) starch (moisture content in the raw material: 10 wt%)—density: 1.48 g·cm^−3^. Generally, this is an unusual effect because starch, under high-temperature conditions, tends to create air pores, which can naturally limit the density of such materials [42]. However, this effect could be the reason for limited access to water, which probably made it impossible to melt all the starch granules (starch granules visible in Figure 5c). The partially melted potato starch therefore served as a binder that supported the precise compression of the sample. Additionally, a small amount of water and a temperature of 140 °C enabled the water to evaporate quickly (while maintaining a constant stress during forming of 20 MPa for 4.5 min). Under these process conditions, the intensity of water evaporation was low and pore formation was very limited.

Conversely, increasing the moisture content in the raw material intensified water evaporation during the production of the biocomposite. This probably resulted in a more complete melting of the starch, but at the same time it contributed to the formation of small air voids throughout the material, which naturally reduced the density of the resulting biocomposites. The obtained density test results can also complement the interpretation of the results of axial strength tests and SEM microscopic images.

### 3.3. Water Contact Angle

The analysis of the wetting angle of the obtained biocomposites revealed that all samples with (SP) starch addition (up to 30%) constituted hydrophilic materials (their wetting angle did not exceed (0° < θ < 90°)) (Figure 4d). On the other hand, further increases in the share of (SP) starch resulted in the limit (>90°) being exceeded, which, according to Grylewicz et al. (2019) [43], indicates a decrease in the hydrophilicity of these materials. The highest wetting angle values (104°) were observed at the starch share of 40 wt% and mixture moisture content of 14%. Example photos showing a drop placed on the surface of biocomposites are shown in Figure 5a–c. These properties can be justified by the better melting of starch in conditions of increased material moisture up to 14%. For example, a wetting angle in the range of 117–120° was obtained in the study of thermoplastic starch by Bastos et al. (2009) [44]. It can therefore be concluded that the addition of starch to extruded apple pomace decreases hydrophilicity of the biocomposites obtained, even without the addition of commonly used plasticisers. It was also observed that biocomposites obtained from apple pomace alone without the addition of starch were characterised by a wetting angle of 45°, which indicates that it is a hydrophilic material, as pointed out by researchers such as Andrade et al. (2005) [45]. This high hydrophilicity of apple pomace may be related to its high content of soluble pectin, representing one of the biocomposite matrix elements. This only highlights the fact that apple pomace should be combined with other additives to decrease the hydrophilicity of such materials. For example, in Lang et al.’s work (2022) [46], the water contact angle of apple pomace plates was 85°, while the water contact angle of plates coated with a special additive was 137°. The results obtained are promising and indicate that it is possible to produce a biodegradable material exclusively from plant-based raw materials with reduced hydrophilic properties. Although the results obtained in the studies only slightly exceed the wetting angle values, this opens up new possibilities to refine biodegradable products exclusively with natural methods. In light of other studies into natural biodegradable materials, the most hydrophobic material has a wetting angle of 158° [47]. In the case of the popular PLA, the wetting angle is only 64° [48]. This explains why biodegradable materials are readily used as a structural material for, e.g., disposable tableware, etc. The results were obtained 1 s after applying the water drops. In order to extend the obtained results, a special coefficient for rough materials presented in article [49] can also be used. This coefficient did not cover the scope of tests performed. Further research presented in the “Moisture Absorption” section showed that the obtained biocomposites absorb water during several hours of exposure to a humid environment. 

### 3.4. Colour Analysis

**Figure 6:**
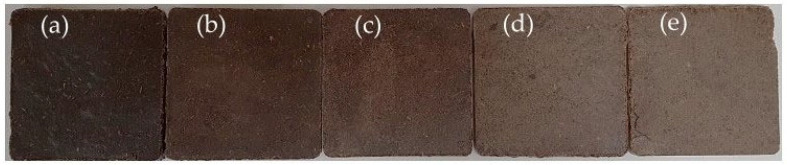
Biocomposites from extruded apple pomace (EAP) and (**a**)—0, (**b**)—10, (**c**)—20, (**d**)—30, and (**e**)—40% addition of potato starch (SP). Moisture content in the raw material: 12%.

The colour analysis performed showed that the samples obtained were characterised by a dark colour grading towards shades of brown and violet—Figure 4e–g. This is directly related to values of the a* and b* parameters, which range from −1.01 to 3.93 in the L*a*b* colour space. Figure 4f shows images of the produced biocomposite samples. It was furthermore found that values of the a* factor increased with the share of (SP) starch up to 30 wt% and then decreased strongly. As this phenomenon is observed over the entire moisture range, it may be related to the intensely progressive non-enzymatic browning reaction (Maillard reaction) of melted starch in particular, as well as pectin and dietary fibre [50]. It may also be related to the migration of water and, at the same time, liquid gel to the sample surface [51], where it is exposed to direct contact with mould elements heated up to 140 °C. The above claim is confirmed by the observed changes in L* brightness. From a technological point of view, such a phenomenon may favourably influence the formation of the internal and external structure of the biocomposite, resulting in a smooth surface. In this case, the water (bound in the extruded gel) evaporates quickly from outer parts of the material, resulting in reduced boiling within the material structure [52]. This can therefore significantly reduce internal porosity of the biocomposites. Figure 6 below shows, as an example, samples of biocomposites made from apple pomace with added starch. The dark colour of the obtained samples is characteristic of many kinds of thermally treated fruit pomace [53].

### 3.5. Scanning Electron Microscope (SEM)

**Figure 7:**
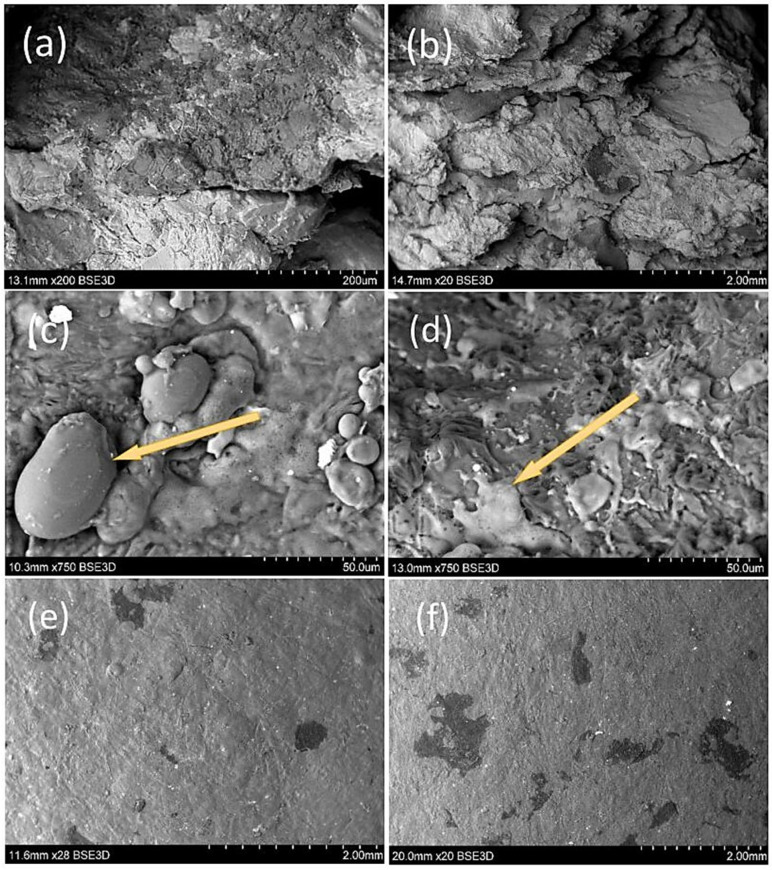
SEM scanning microscopy images of the biocomposites: (**a**,**b**)—“breakthrough” of the sample; (**c**)—starch granules of non-gelatinised starch (indicated by the arrow); (**d**)—gelatinised starch (indicated by the arrow); (**d**–**f**)—surface area comparisons of samples produced at 10 and 14% raw material moisture. Samples (**a**–**d**): starch addition—40%, raw material moisture—10%.

SEM microscopic photographs were taken to investigate the internal and external structure of the fabricated biocomposites (Figure 7a–f). The utilitarian aim was to identify characteristic features of the biocomposite structure that could have an indirect effect on cracking of the samples during strength tests. The SEM microscopic analysis showed that samples produced from extruded apple pomace were characterised by an irregular structure, which consisted of agglomerated material particles combined with melted starch and pectin gel, as can be observed in Figure 7d. A characteristic feature of these materials was also found to be flattened material particles to varying extents, as shown in Figure 7a. In addition, various elongations can be observed at sample fracture surfaces, which are indicative of a violation of the consistency of the biocomposite and pulling out of the fibres due to mechanical force [25]. Sample consistency, on the other hand, could be affected by hydrophilic or hydrophobic reactions as indicated by Mofokeng et al. (2012) [54]. Meanwhile, increasing the share of (SP) starch in the sample could result in improved interfacial interactions, which could result in better energy dissipation during sample breakage [55]. In the analysed samples produced from a raw material with a moisture content of 10–14%, starch granules embedded in the gel could also be observed (Figure 7c). The observed granules are unmelted starch in samples from raw material with a moisture content of 10%, or resistant starch. Starch granules show flattening, indicating direct contact between the starch and the matrix heated to 140 °C. This may indicate a lack of availability of the water necessary to melt the starch. A comparison of the surface of samples refined with the addition of starch (40%) with those made at 10 and 14% moisture content showed that a smoother surface was characteristic of the samples made from the raw material with the higher moisture content.

### 3.6. Thermogravimetry Analysis (TGA) and Derivative Differential Thermal Analysis (DTA)

**Figure 8:**
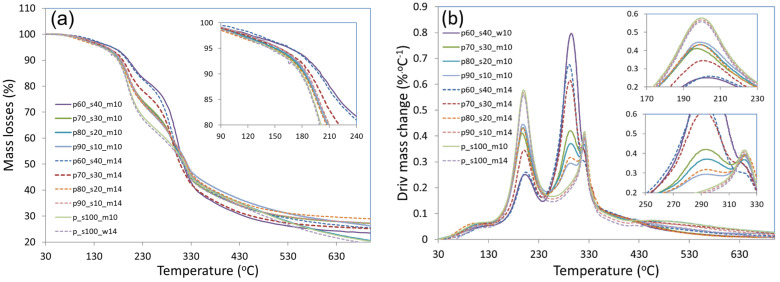
The course of (mass losses) (**a**) and (derivative mass change biocomposites) (**b**) curves of biocomposites from (EAP) extruded apple pomace and (SP) starch.

Table 3 shows the percentage mass loss for each temperature range during the thermogravimetric analysis. Figure 8a,b then show the exact progression of the TGA and DTA analysis. The diagrams present the five main stages of mass loss. In the first zone of 30–150 °C, which illustrates mainly water evaporation, the weight loss was 2.93–4.22% and was proportionally lower for samples that were prepared from a raw material with a moisture content of 14%. In the second range of 150–220 °C, the weight loss ranged from 15.79 to 32.09% and decreased with the share of starch in the biocomposite. Thus, one can conclude that, in this case, the addition of starch was crucial for temperature resistance of the materials tested. In this temperature range, the degradation of low-molecular-weight components and initial degradation (softening) of cellulose are usually responsible for the weight loss [56]. Weight loss found in the 250–350 °C temperature range could be related to the degradation of polysaccharides contained in the sample. For example, according to Lin et al. (2007) [57], cellulose degradation usually occurs in this temperature range. The further degradation of the material in the temperature range of 350–600 °C is related to the acetylated degradation of high-molecular-weight components, depolymerisation, and other transformations as indicated by Yang et al. (2007) [58,59]. The analysis carried out showed that the first 5% weight loss occurred at 142.1 °C, which was observed for the sample with a 20% starch addition (raw material moisture content of 10%). In contrast, the latest 5% weight loss was observed for samples with a 40% starch addition. In this case, weight loss was observed at 166.5 °C (sample made from raw material with a 10% moisture content) and 171.4 °C for the sample made from raw material with a 14% moisture content. Weight loss of 50% usually occurred between 301.1 and 330.1 °C and was lowest for samples with 40% starch for the two extremes of raw material moisture content used.

### 3.7. FTIR Infrared Spectrum Analysis

**Figure 9:**
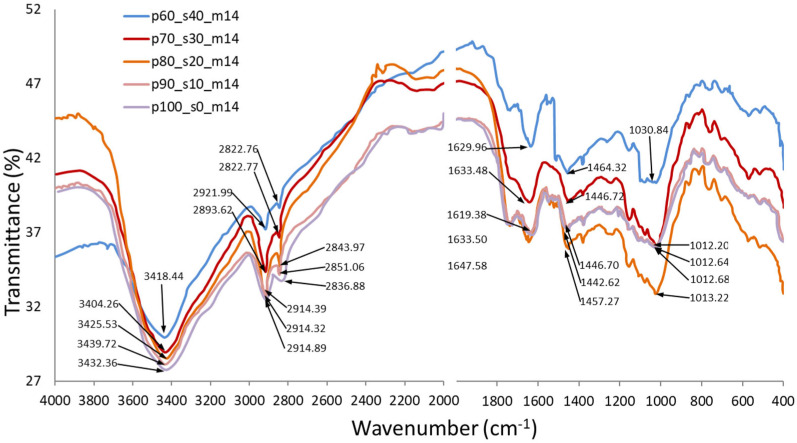
FTIR infrared spectrum of biocomposites from extruded apple pomace (EAP) for 40, 30, 20, 10, and 0% addition of potato starch (SP). Moisture content in the raw material: 14%.

Figure 9 shows the spectra of FTIR spectrum analyses performed for biocomposite samples obtained from raw material with a 14% moisture content. All spectra were found to have a typical waveform and represent characteristic vibrational types for biological materials rich in dietary fibre. It was also observed that all spectra had a similar waveform, with variations mainly involving a slight shift in spectral transmittance distribution. When observing the individual spectra, three main areas can be distinguished: The first area is in the range of 3450–3000 cm^−1^, indicating the presence of stretching vibrations of the 0-H methyl group bonds. The second area occurs in the range of 2200–1500 cm^−1^ and indicates the presence of compounds containing double bonds (C=O, C=C) [60]. For example, Nagasaki et al. estimated the absorbance at 1640 cm^−1^ increasing with increasing humidity [61]. The third area includes the fingerprint region, where bands originate from single C-O bonds and bands responsible for deformational vibrations [62]. When analysing the results obtained, it can also be seen that the transmittance value increases with an increase in starch addition, with the highest values achieved for hydroxyl (-OH) bonds. The analyses carried out also showed that the transmittance value was higher for biocomposites obtained from raw material with a moisture content of 10%.

### 3.8. Moisture Absorption

**Figure 10:**
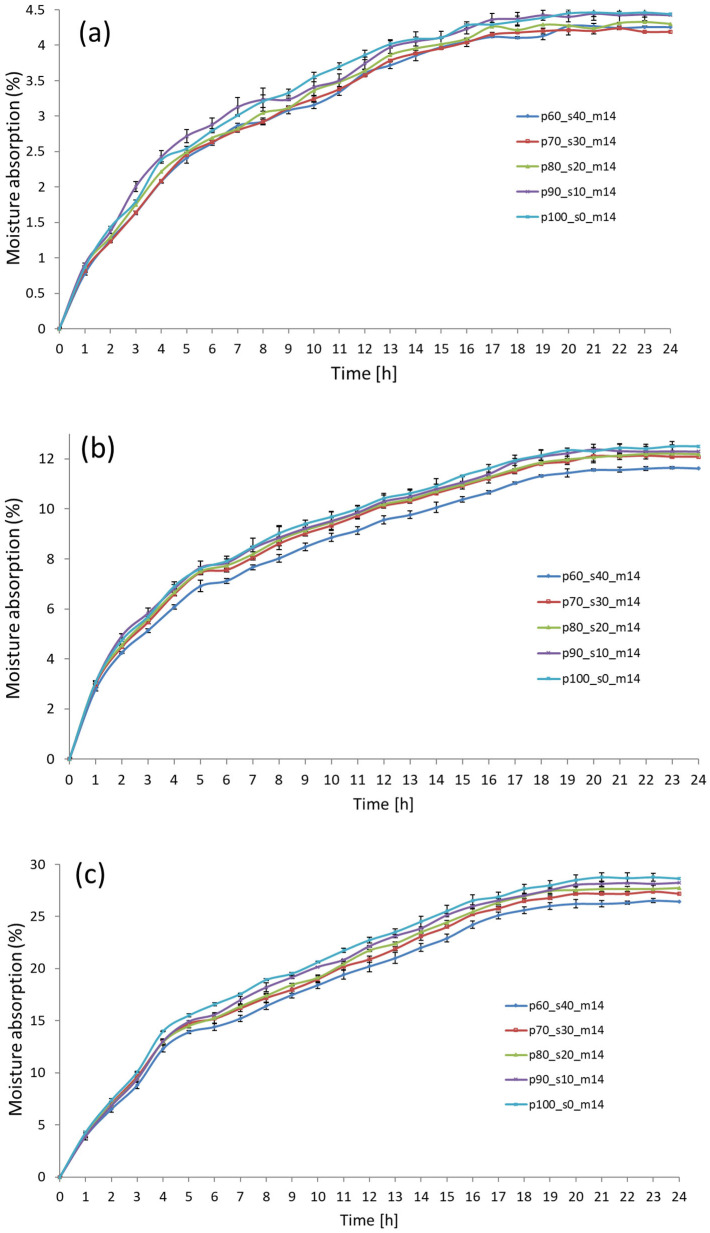
Course of changes in absorption of moisture from air by biocomposites. (**a**) Relative humidity, RH—50%; (**b**) relative humidity, RH—75%; (**c**) relative humidity, RH—90%.

Moisture absorption tests (MA) performed in a climatic chamber showed that the samples were sensitive to changes in air humidity (Figure 10a–c). It was found that the samples became saturated with moisture within 20 h, with the most intense increase in the water absorption of the samples observed in the first four hours. During the remaining hours of the test, the samples stabilised and only minor changes in moisture absorption were noticeable. It can also be stated that the water absorption of the samples increased with a decrease in the EAP content of the samples, which could be observed at all climatic conditions used (RH, 50, 75, and 90%). Thus, it can be concluded that samples p60_s40_m10 had the highest water absorption, and samples p100_s0_m10 had the lowest. It was also found that the samples tested at a relative humidity of 50% RH were the most sensitive to moisture changes. In this case, the water absorption after 24 h of the samples being in the chamber was only 4.53%, but the increase in water absorption during the first four hours was as much as 50–55% of the total water absorption of the samples (Figure 10a). In the case of samples exposed to air humidity conditions RH 75 and 90%, the course of air moisture absorption was more uniform, but the absorbability of the samples was significantly higher. The highest average water absorption recorded for samples p60_s40_m10 was 12.24% (RH 75%) and 28.55% (RH 90%). Generally, this behaviour of the samples can be explained mainly by the increased share of (SP) starch in the biocomposite structure. Starch melts during thermal treatment, and then the water contained in the product evaporates intensively, resulting in a change in the pressure difference in the matrix for forming biocomposites. A similar process occurs in the extrusion process [63]. In this way, a product is created with low water activity, which is water-absorbing and at the same time sensitive to changes in air humidity [64,65]. Moreover, extruded EAP pomace may also contain trace amounts of starch, which further intensifies the entire process [66]. Another reason is the hydrophilic nature of lignocellulosic components, which are also found in EAP [67]. For comparison, biocomposites made from pomace such as residues from the processing of carrots, oranges, or spinach, at maximum air saturation with water vapor RH 100%, absorbed up to 40–60% of moisture from the air [68]. Referring to the results obtained and the research of other authors, it can be concluded that materials made from extruded (EAP) apple pomace and (SP) starch should not be subjected to hourlong exposure to high-air-humidity conditions, RH 75, 90%. However, sensitivity to increased air humidity may be a positive feature of these biocomposites because the increased water absorption of these products may accelerate the biodegradation process initiated in atmospheric conditions. This may also be a positive feature for the previously mentioned pots and multiplates, which, after use, could be easily converted into compost for fertilising plants. This may also indirectly reduce the production costs of composite materials, which are influenced by the price of electricity, starch, and, to a lesser extent, apple pomace. However, this was not the subject of our research.

## 4. Conclusions

Research has shown that it is possible to produce a biocomposite from (EAP) apple pomace reinforced with (SP) potato starch. Moreover, the produced biocomposite materials were easy to press, were reproducible, and were of good quality. It was found that the pomace processed by extrusion in combination with the starch allows the production of a biocomposite with a compact structure and a smooth surface, as shown by SEM microscopic images. The colour of extruded apple pomace biocomposites is dark brown and brightens with growing starch content. Furthermore, it was found that the addition of (SP) potato starch in each case contributed to improving strength parameters of the biocomposites obtained. This study showed that the strongest materials were the biocomposites produced with the highest share of (SP) starch and with extreme moisture contents of 10% and 14%. Density tests have shown that the addition of (SP) potato starch does not necessarily reduce the density of the produced biocomposites. This may suggest that two technologies can be adopted in the manufacture of such biodegradable materials. The first is the “dry” one, where materials are bonded mainly by pressure and the van der Waals forces present in the material. In this case, unmelted starch can act as a natural reinforcement, with some of the melted starch and pectin gel providing an additional natural binder. The other technology, promoting an increase in moisture content, where the vast majority of the starch is melted and the pectin presumably interacts with the gel, also contributes to the strengthening of the biocomposite. The advantage of the second method lies in obtaining a smoother surface using a moisture content of 14%. On the other hand, better temperature resistance will be obtained by manufacturing the product from raw material with a moisture content of 10%, as confirmed by the TGA tests. Next, FTiR studies reveal that the transmittance value is higher for biocomposites obtained from raw material with a moisture content of 10%. The hydroxyl bond values also increase with the addition of starch. Finally, air moisture absorption studies showed that EAP and SP biocomposites are sensitive to relative air humidity, in particular RH 75 and 90%. The research shows that extruded apple pomace combined with potato starch is a promising raw material that can be used to produce biodegradable materials that do not require a high stress transfer, e.g., household items, plates, cups, etc. This type of products can also be used in agriculture to produce products such as flower pots, pot pallets, pads, etc. This would undoubtedly limit the use of plastics in the agri-food industry. For a wider application of apple pomace, however, further development of this technology towards the search for even more advanced biodegradable additives is necessary.

## Figures and Tables

**Table 1:** Empirical test plan.

Sample Number	Index *	Apple Pomace (EAP) [wt%]	Starch [wt%]	Moisture [%]
1	p60_s40_m10	60	40	10
2	p70_s30_m10	70	30	10
3	p80_s20_m10	80	20	10
4	p90_s10_m10	90	10	10
5	p100_s0_m10	100	0	10
6	p60_s40_m12	60	40	12
7	p70_s30_m12	70	30	12
8	p80_s20_m12	80	20	12
9	p90_s10_m12	90	10	12
10	p100_s0_m12	100	0	12
11	p60_s40_m14	60	40	14
12	p70_s30_m14	70	30	14
13	p80_s20_m14	80	20	14
14	p90_s10_m14	90	10	14
15	p100_s0_m14	100	0	14

*—indexes used for TGA, DTA, and FTiR charts: p—extruded apple pomace, s—potato starch, m—moisture.

**Table 2:** Anova results, SS: sum of squares, df: degree of freedom, MS: sum of mean squares, F: statistics, *p:* value of test probability, L: linear, Q: square.

Source of Variation		Bending Strength (MPa); R^2^= 0.762; Pure error MS = 0.848				
		SS	df	MS	F	*p*
1, Moisture (%)	L	12.908	1	12.908	15.218 *	0.0005
Moisture (%)	Q	8.500	1	8.500	10.022 *	0.0035
2, Starch (wt%)	L	74.721	1	74.721	88.093 *	0.0000
Starch (wt%)	Q	5.800	1	5.800	6.838 *	0.0138
Interaction of 1 L vs. 2 L		4.375	1	4.375	5.158 *	0.0305
Lack of fit		7.713	9	0.857	1.010	0.4537
Pure error		25.446	30	0.848		
Total SS		139.463	44			
**Young’s modulus (MPa); R^2^= 0.871; Pure error MS = 00036**						
1, Moisture (%)	L	0.002	1	0.002	5.509 *	0.0257
Moisture (%)	Q	0.027	1	0.027	74.898 *	0.0000
2, Starch (wt%)	L	0.565	1	0.565	1573.495 *	0.0000
Starch (wt%)	Q	0.050	1	0.050	138.238 *	0.0000
Interaction of 1 L vs. 2 L		0.005	1	0.005	14.932 *	0.0006
Lack of fit		0.086	9	0.001	26.562 *	0.0000
Pure error		0.011	30	0.0004		
Total SS		0.74	44			
**Density (g·cm^−1^); R^2^ = 0.712; Pure error MS = 0.000839**						
1, Moisture (%)	L	0.0491	1	0.0491	58.4716 *	0.0000
Moisture (%)	Q	0.000001	1	0.000001	0.00138	0.9706
2, Starch (wt%)	L	0.0111	1	0.0111	13.2115 *	0.0010
Starch (wt%)	Q	0,0011	1	0.0011	1.2858	0.2658
Interaction of 1 L vs. 2 L		0.0175	1	0.017	20.8718 *	0.0001
Lack of fit		0.0246	9	0.0027	3.2555 *	0.0071
Pure error		0.0252	30	0.0008		
Total SS		0.1286	44			
**Water contact angle (°); R^2^ = 0.881; Pure error MS = 0.866**						
1, Moisture (%)	L	173.761	1	173.761	220.634 *	0.0000
Moisture (%)	Q	67.254	1	67.254	85.396 *	0.0000
2, Starch (wt%)	L	6201.760	1	6201.760	7874.695 *	0.0000
Starch (wt%)	Q	333.206	1	333.206	423.089 *	0.0000
Interaction of 1 L vs. 2 L		231.673	1	231.673	294.168 *	0.0000
Lack of fit		924.457	9	102.717	130.426 *	0.0000
Pure error		23.627	30	0.788		
Total SS		7955.739	44			
L*; R^2^= 0.732; Pure error MS = 0.055						
1, Moisture (%)	L	0.702	1	0.702	2.330	0.1366
Moisture (%)	Q	18.432	1	18.432	61.457 *	0.0000
2, Starch (wt%)	L	30.769	1	30.769	102.592 *	0.0000
Starch (wt%)	Q	1.165	1	1.165	3.886	0.0579
Interaction of 1 L vs. 2 L		8.853	1	8.853	29.517 *	0.0000
Lack of fit		29.492	9	3.276	10.926 *	0.0000
Pure error		8.997	30	0.299		
Total SS		98.411	44			
a*; R^2^= 0.719; Pure error MS = 0.00095						
1, Moisture (%)	L	0.197	1	0.197	206.465 *	0.0000
Moisture (%)	Q	0.188	1	0.188	196.878 *	0.0000
2, Starch (wt%)	L	5.730	1	5.730	6011.003 *	0.0000
Starch (wt%)	Q	8.265	1	8.265	8669.272 *	0.0000
Interaction of 1 L vs. 2 L		0.045	1	0.045	47.596 *	0.0000
Lack of fit		5.602	9	0.622	652.913 *	0.0000
Pure error		0.029	30	0.00095		
Total SS		20.056	44			
b*; R2= 0.711; Pure error MS = 0.0065						
1, Moisture (%)	L	0.853	1	0.853	130.809 *	0.0000
Moisture (%)	Q	1.439	1	1.4394	220.546 *	0.0000
2, Starch (wt%)	L	30.543	1	30.543	4681.378 *	0.0000
Starch (wt%)	Q	0.761	1	0.761	116.587 *	0.0000
Interaction of 1 L vs. 2 L		0.111	1	0.111	17.004 *	0.0003
Lack of fit		13.479	9	1.498	229.550 *	0.0000
Pure error		0.196	30	0.0065		
Total SS		47.382	44			

*—Significant at *p* ≤ 0.05.

**Table 3:** Summary of percentage mass loss during TGA analysis.

Sample	Mass Losses (%)					Temperature at 5% Mass Losses (°C)	Temperature at 50% Mass Losses (°C)
	I 30–150 °C	II 150–250 °C	III 250–350 °C	IV 350–600 °C	Total 30–600 °C		
p60_s40_m10	3.37	15.79	54.15	0	73.31	166.5	314.1
p70_s30_m10	4.02	24.28	20.91	22.49	71.70	153.2	322.7
p80_s20_m10	4.22	24.77	18.85	29.13	76.97	142.1	325.6
p90_s10_m10	3.55	26.39	14.75	26.64	71.33	158.5	330.1
p60_s40_m14	3.19	15.64	54.49	0	73.32	171.4	301.1
p70_s30_m14	2.93	20.41	49.91	0	73.25	158.5	315.5
p80_s20_m14	4.09	25.17	16.75	24.52	70.53	150.1	329.7
p90_s10_m14	3.71	25.69	15.88	26.30	71.58	159.3	328.1
p100_s0_m10	4.15	32.09	33.75	0	69.99	149.3	325.9

p—extruded apple pomace, s—potato starch, m—moisture.

## Data Availability

The original contributions presented in the study are included in the article, further inquiries can be directed to the corresponding author.
